# Polyunsaturated fatty acid-based targeted nanotherapeutics to enhance the therapeutic efficacy of docetaxel

**DOI:** 10.1080/10717544.2017.1373163

**Published:** 2017-09-09

**Authors:** Thiruganesh Ramasamy, Pasupathi Sundaramoorthy, Hima Bindu Ruttala, Yongjoo Choi, Woo Hyun Shin, Jee-Heon Jeong, Sae Kwang Ku, Han-Gon Choi, Hwan Mook Kim, Chul Soon Yong, Jong Oh Kim

**Affiliations:** a College of Pharmacy, Yeungnam University, Gyeongsan, Republic of Korea;; b Department of Medicine, Center for Ultrasound Molecular Imaging and Therapeutics, University of Pittsburgh, Pittsburgh, PA, USA;; c Gachon Institute of Pharmaceutical Sciences, Gachon University, Incheon, Republic of Korea;; d Division of Hematologic Malignancies & Cellular Therapy, Duke University Medical Center, Durham, NC, USA;; e College of Korean Medicine, Daegu Haany University, Gyeongsan, Republic of Korea;; f College of Pharmacy, Institute of Pharmaceutical Science and Technology, Hanyang University, Ansan, Republic of Korea

**Keywords:** Docetaxel, folic acid, nanotherapeutics, polyunsaturated fatty acids, synergy

## Abstract

Since breast cancer is one of the most lethal malignancies, targeted strategies are urgently needed. In this study, we report the enhanced therapeutic efficacy of docetaxel (DTX) when combined with polyunsaturated fatty acids (PUFA) for effective treatment of multi-resistant breast cancers. Folic acid (FA)-conjugated PUFA-based lipid nanoparticles (FA-PLN/DTX) was developed. The physicochemical properties, *in vitro* uptake, *in vitro* cytotoxicity, and *in vivo* anticancer activity of FA-PLN/DTX were evaluated. FA-PLN/DTX could efficiently target and treat human breast tumor xenografts *in vivo*. They showed high payload carrying capacity with controlled release characteristics and selective endocytic uptake in folate receptor-overexpressing MCF-7 and MDA-MB-231 cells. PUFA synergistically improved the anticancer efficacy of DTX in both tested cancer cell lines by inducing a G2/M phase arrest and cell apoptosis. Combination of PUFA and DTX remarkably downregulated the expression levels of pro-apoptotic and anti-apoptotic markers, and blocked the phosphorylation of AKT signaling pathways. Compared to DTX alone, FA-PLN/DTX showed superior antitumor efficacy, with no signs of toxic effects in cancer xenograft animal models. We propose that PUFA could improve the therapeutic efficacy of anticancer agents in cancer therapy. Further studies are necessary to fully understand these findings and achieve clinical translation.

## Introduction

Breast cancer remains one of the most lethal malignancies and the second leading cause of cancer-related deaths in women (Wang et al., 2016). In 2015, there were approximately 45,000 deaths due to breast cancer and more than 200,000 new cases were recorded. Despite the significant advancement in chemotherapeutic modality, incidence of cancer mortality is increasing every year (Tang et al., 2015; Jin et al., 2016). Therefore, design and development of anticancer drug-based strategies are a significant priority for effective breast cancer treatment.

Docetaxel (DTX), a microtubule-stabilizing taxane, is one of the most active anticancer agents for treating metastatic breast cancer, and acts via inhibition of cell cycle progression (at mitotic phase) by inducing tubulin polymerization, causing cell cycle arrest at G2/M phase that results in cell death (Jiang et al., 2015). Similar to that for most anticancer drugs, clinical application of DTX is hindered due to its poor water solubility, nonselective distribution/targeting, and rapid systemic clearance, which critically affect its therapeutic efficacy (Ren et al., 2016). Besides, DTX resistance remains a major concern in successful cancer treatment. In order to overcome these limitations, numerous DTX-loaded nanoformulations have been developed, such as micelles, polymeric nanoparticles (NPs), liposomes, SMEDDS, and lipid-emulsified NPs (Zhao & Astruc, 2012; Guo & Huang, 2014; Jain et al., 2016; Zhu et al., 2016; Kim, 2016). Notably, Cellax^TM^ (DTX covalently conjugated with polyethylene glycol-acetylated carboxymethylcellulose, which forms self-assembled 120 nm particles) and BIND-014 (DTX encapsulated in biodegradable polymer-based NPs) have emerged as clinically useful nanoformulations (Zhao & Astruc, 2012; Guo & Huang, 2014). However, the applications of these formulations are limited due to complicated preparation protocols, limited stability, and heterogeneous nature of cancer cells among others (Marusyk et al., 2012; Shi et al., 2015; He et al., 2015; Choi et al., 2016; De Pauw et al., 2016; Pawar et al., 2016; Lee & Youn, 2016; Sarisozen et al., 2017). Therefore, alternative strategies need to be developed to improve the chemotherapeutic efficacy of DTX while also reducing the associated side effects.

Polyunsaturated fatty acids (PUFA) have attracted increasing attention owing to their ability to inhibit the progression of many cancers and induce cell apoptosis (Berquin et al., 2008; Bougnoux et al., 2010; Siddiqui et al., 2011). PUFA have been used to improve the oral bioavailability of small molecules, such as vancomycin (Kajita et al., 2000). For example, Amiji et al. (Tiwari & Amiji, 2006; Vyas et al., 2008) showed that chemotherapeutic efficacy of paclitaxel (PTX) and saquinavir increased when administered with PUFA in a microemulsion form. Recently, taxoprexin, a conjugate of PTX with docosahexaenoic acid (a PUFA), was synthesized. Taxoprexin enhanced the therapeutic activity and greatly decreased the systemic toxicity of PTX (Roy et al., 2015). Among various PUFA, γ-linolenic acid (GLA) is unique because of its potential to inhibit inflammatory responses, metastasis, and apparently tumor growth (Cao et al., 2016). Several *in vitro* studies have reported the anticancer effect of GLA in leukemia and breast, brain, pancreas, and colon cancers (Ribeiro et al., 2015). PUFA are preferentially taken up by tumor cells and are therefore used as tumor-specific ligands to guide antitumor drugs to recognize tumor cells selectively (Xu & Qian, 2014), highlighting the need for an approach that has the capability to differentiate between various cell types present in the tumor microenvironments.

Nanomedicines are an attractive approach of antitumor drug delivery and hold a great potential in cancer treatment. Owing to their size and surface properties, NP can increase the therapeutic efficacy and lower the associated side effects of anticancer agents by accumulating in tumor tissues due to enhanced permeation and retention (EPR) effect (Ruttala et al., 2017; Ramasamy et al., 2017). To increase the specificity of NP, folic acid (FA) has been reported to be an effective targeting moiety that can selectively bind to folate receptors (FRs), which are overexpressed in many primary and metastatic tumors but have negligible presence in normal cells (Leamon & Reddy, 2004; Liu et al., 2009). The FA containing NP could bind to FRs with high affinity via a typical receptor-mediated endocytosis mechanism.

Therefore, in this study, novel PUFA-based lipid NP comprising PUFA, D-α-tocopheryl polyethylene glycol 1000 succinate (TPGS), and FA were developed to improve the chemotherapeutic efficacy of DTX. The efficacy of the nanoformulations was investigated by various *in vitro* and *in vivo* experiments. Moreover, the role of PUFA was investigated through a detailed mechanistic signaling pathway analysis. The synergistic potential of PUFA for improving the therapeutic efficacy of DTX was studied in a metastatic breast cancer model.

## Methods

DTX was obtained from Taihua Co (Xi’an, China). Tricaprin was purchased from TCI Chemicals (Seoul, South Korea). γ-linolenic acid (GLA), lecithin, Tween 80, stearic acid (SA), FA, D-α-tocopheryl poly(ethylene) glycol 1000 succinate (TPGS), 1-ethyl-3-(3-dimethylaminopropyl) carbodiimide (EDC), *N*-hydroxysulfosuccinimide (NHS), and dimethylformamide (DMF) were purchased from Sigma-Aldrich (St Louis, MO). All other chemicals were of reagent grade and used without further purifications.

### Synthesis of FA–SA conjugates

An FA**–**SA conjugate was synthesized as reported earlier. Briefly, 200 mg SA was dissolved in DMF followed by addition of EDC/NHS and reaction for 2 h. FA (30 mg) dissolved in pyridine was then added to SA solution and reacted overnight in the dark. FA**–**SA conjugate was separated by addition of ice-cold water and the obtained product was dialyzed (molecular weight cut off 8000) against distilled water to remove the unreacted components. The product was collected by freeze drying and stored in the refrigerator. The product was confirmed using ^1^H NMR and FTIR (Supplementary Figure S1).

### Preparation of FA-conjugated lipid NPs

Tricaprin (100 mg), lecithin (10 mg), TPGS (5 mg), GLA (3 mg), and FA**–**SA (3 mg) were added in a glass tube and melted at 75 °C (lipid melt). DTX (10% w/w) was added to the lipid melt and stirred to dissolve. An aqueous solution of Tween 80 (2%) was heated to the same temperature and transferred to the above mixture. The mixture was immediately homogenized at 14,000 rpm for 5 min (T25 ULTRA-TURRAX, IKA, Staufen, Germany) and probe sonicated (Vibracell VCX130; Sonics, Newtown, CT) for 3 min at 80 W to prepare NPs. The obtained NP suspension was then dialyzed against distilled water for 12 h to remove free drug or reaction materials and stored at 4 °C until further use.

### Loading capacity and loading efficiency

The loading capacity and efficiency were evaluated by centrifugation method. The DTX-loaded FA-PLN (FA-PLN/DTX) were placed in an Amicon centrifugal filter device (MWCO 10000 Da, Millipore) and centrifuged at 5000 rpm for 15 min. The filtrate was collected and subjected to HPLC analysis to determine the drug loading capacity of NPs. The HPLC system (Hitachi, Tokyo, Japan) consisted of a pump (Model L2100), an auto sampler (Model L2200), and an ultraviolet detector (Model L2420). A C_18_ column (Inertsil ODS3: 0.5 μm, 15 × 0.46 cm, GL Sciences Inc., Japan) was used. Acetonitrile and phosphate buffer (pH 5) at a volume ratio of 49/51 was used as the mobile phase and effluent was detected at 232 nm.

### Particle characterization

The average particle size, polydispersity index (PDI), and zeta potential were evaluated using Zeta Sizer Nano ZS (Malvern Instruments), which uses the principles of dynamic light scattering (DLS) for measurements. Measurements were performed at room temperature at a fixed angle of 90°. For morphological analysis, a drop of NPs was deposited on a copper grid, counter stained with 2% (w/v) phosphotungstic acid, and then air-dried. The morphology of NPs was visualized using a transmission electron microscope (TEM; H-7600, Hitachi, Tokyo, Japan) at an accelerating voltage of 100 kV. The crystalline state of drug-loaded NPs was determined using an X-ray diffractometer (X’Pert PRO MPD diffractometer, Almelo, the Netherlands) equipped with a copper anode (Cu Kαradiation) at 40 kV and 30 mA. Differential scanning calorimetry (DSC) experiments were performed using the DSC-Q200 calorimeter (TA Instruments, New Castle, DE) at a heating rate of 10 °C/min from 40 °C to 250 °C. The FTIR spectra were recorded using a Bomen MB-II FTIR spectrometer (Hartmann & Brawn Co).

### *In vitro* drug release study

*In vitro* drug release experiment was performed using the dialysis method. Briefly, 1 mL of nanoformulations (2 mg of equivalent DTX) was placed in a dialysis membrane (Spectra/Por; MWCO 3,500 Da). The samples were immersed in phosphate buffered saline (pH 7.4) and acetate buffered saline (pH 5.0) and placed in a shaking water bath. At specified intervals, 1 mL buffer was withdrawn and replaced with equal volume of fresh release buffer. The amount of drug released at each time point was determined by HPLC method as described above.

### Cell culture

MCF-7 and MDA-MB-231 breast cancer cells were grown in Roswell Park Memorial Institute Media (RPMI) containing 10% fetal bovine serum and 1% penicillin/streptomycin at 37 °C in a humidified atmosphere containing 5% CO_2_.

### Cytotoxicity assay

*In vitro* cytotoxicity of DTX, GLA, PLN/DTX, and FA-PLN/DTX in MCF-7 and MDA-MB-231 cells was evaluated by MTT assay. The cells were seeded in 96-well plates and exposed to respective formulations for 24 h. MTT solution (10 µL, 5 mg/mL) was then added to the cells and plates were incubated for 4 h. Subsequently, 100 µL of DMSO was added to each well and absorbance was read after 15 min using a microplate reader at 570 nm.

### Quantitative cellular uptake analysis

Cells (2 × 10^5^) were seeded in a six-well plate and grown for 24 h. The cells were incubated with PLN/DTX (non-targeting) and FA-PLN/DTX (targeting) nanoformulations for 2 h. Rhodamine-B was used as a fluorescent probe. After treatment, the cells were washed twice and detached by trypsinization. The cells were then centrifuged and the obtained cell pellets were resuspended in PBS followed by analysis using FACS Calibur^TM^ flow cytometer. The mean fluorescence intensity (MFI) was analyzed at excitation wavelength of 495 nm and emission at 519 nm.

### Intracellular uptake

MCF-7 and MDA-MB-231 cells (2 × 10^5^) were seeded on cover slips placed in six-well plates and incubated for 24 h. The cells were incubated with PLN/DTX and FA-PLN/DTX nanoformulations for 2 h at 37 °C. After treatment, the cells were fixed with 4% paraformaldehyde for 10 min and washed. Subsequently, the cells were mounted on a glass slide and observed under a confocal laser scanning microscope (Nikon A1+, Japan).

### Apoptosis and cell cycle analysis

First, the apoptotic potential of individual formulation was determined using Annexin V-FITC/PI staining kit (BD Biosciences, NJ). Briefly, MCF-7 and MDA-MB-231 breast cancer cells (2 × 10^5^) were seeded in a six-well plate and incubated for 24 h. The cells were then treated with respective formulations for 24 h. Subsequently, the cells were then washed, collected, and stained with Annexin V-FITC (2 µL) and PI (2 µL) for 15 min. The cells were then analyzed using a FACS Calibur^TM^ instrument. Cells that were Annexin-V^+^ were early apoptotic; Annexin-V^–^/PI^–^ were live cells; whereas Annexin-V^+^/PI^+^ cells were late apoptotic or necrotic.

For cell cycle analysis, cells (2 × 10^5^) were seeded in a six-well plate and incubated for 24 h. The cells were then treated with respective formulations and incubated for 24 h. The cells were collected by trypsinization and fixed with 70% cold ethanol for 1 h. Subsequently, the cells were incubated with 10 µL PI (10 mg/mL) and 5 µL ribonuclease (10 mg/mL) for 30 min at 37 °C in the dark. The cell cycle analysis was performed on the FACS Calibur flow cytometer (BD Biosciences, NJ) (10,000 cells per measurement).

### Western blot analysis

Cells (3 × 10^5^) were seeded in a six-well plate and treated with respective formulations for 24 h. After treatment, the cells were harvested and lysed on ice for 30 min. The whole cell lysate was subjected to sodium dodecyl sulfate-polyacrylamide gel electrophoresis (SDS-PAGE). The electrophoresed samples were blotted onto polyvinylidene difluoride membranes (Millipore). The membranes were blocked with 5% skim milk and then incubated with respective primary antibody overnight at 4 °C. Subsequently, the membranes were washed in TBST buffer and incubated with secondary antibody conjugated to horseradish peroxidase for 2 h. The membranes were then developed for signal detection and imaged using Kodak M35-A X-OMAT processor.

### Antitumor efficacy study

All protocols for animal study were approved by the Institutional Animal Ethics Committee, Yeungnam University, South Korea. Female BALB/c nude mice (7-week-old) were obtained from Orient Bio (Seoul, South Korea) and housed under ambient conditions with free access to food and water. To develop tumors, mice were implanted with 5 × 10^6^ MDA-MB-231 cells/mouse (in 100 µL PBS) in the right flanks. The tumors were allowed to grow until an average tumor volume of 100 mm^3^ was reached. The mice were randomly divided into six groups (seven mice per group). The mice were intravenously administered blank NP (without GLA), DTX, GLA, PLN/DTX, and FA-PLN/DTX at a dose of 5 mg/kg (of DTX) through tail veins. The formulations were administered four times during the first 12 days of the study. Tumor volume of individual mice was determined by vernier caliper using the following formula: tumor volume (mm^3^) = 0.5 × a × b^2^; where ‘a’ represents the length and ‘b’ represents the width of the tumor. Body weight index was also monitored throughout the study period. At the end of the experiment, mice were sacrificed and tumors were collected, embedded in paraffin, and sectioned at 4 µm followed by immunohistochemical analysis.

### Statistical analysis

Data were analyzed by using Student's *t*-test (equal variance) for comparison between two different groups. Results were considered statistically significant at *p* < .05. All data reported are mean value ± SD.

## Results

### Preparation of FA-modified PUFA-based lipid NPs

**Figure 1.**
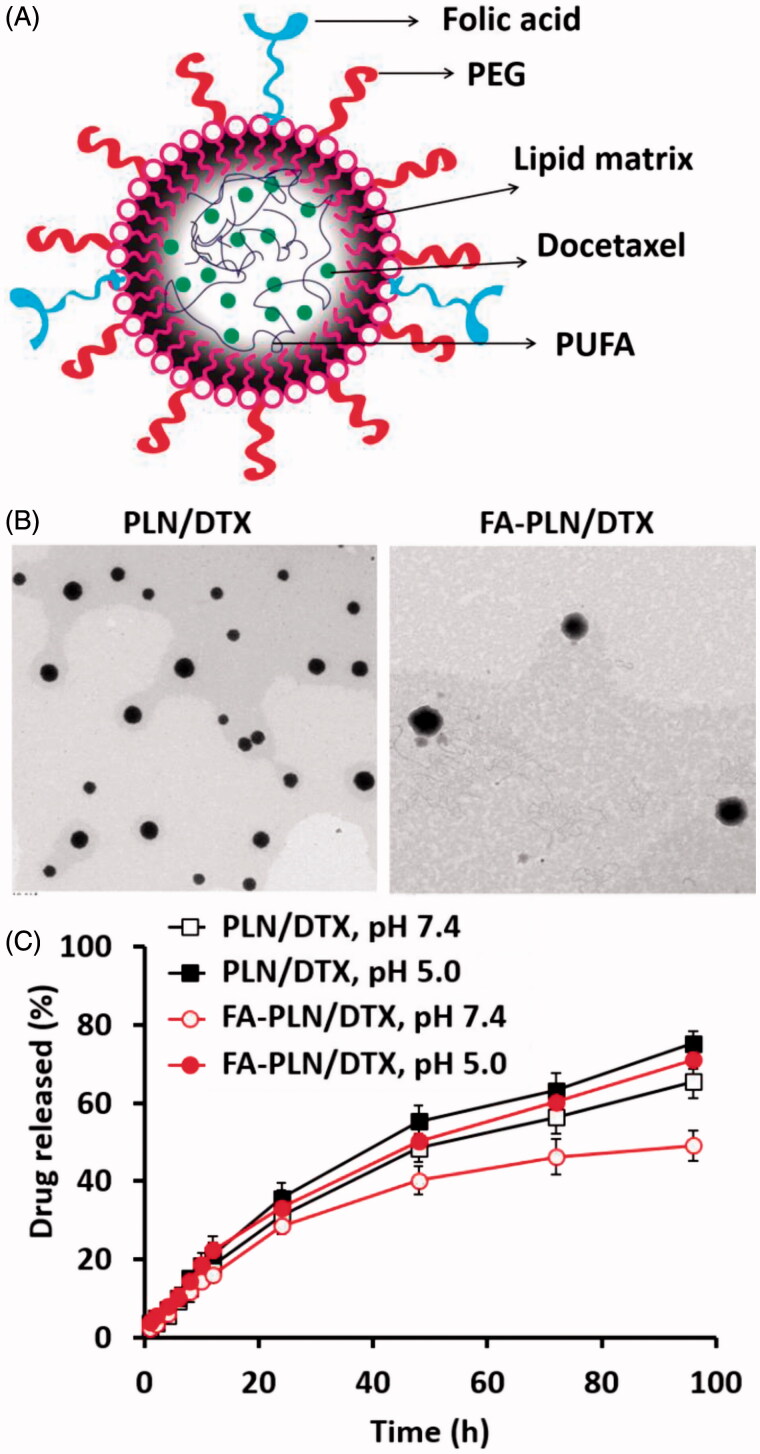
(A) Schematic of preparation of PUFA-based FA-conjugated lipid NPs for breast cancer targeting. (B) Transmission electron microscope (TEM) image of PLN/DTX and FA-PLN/DTX. (C) *In vitro* drug release profile of DTX from PLN/DTX and FA-PLN/DTX in PBS (pH 7.4) and ABS (pH 5.0) at 37 °C (*n* = 3).

In this study, FA-conjugated PUFA-based lipid NPs (FA-PLN/DTX), comprising GLA, TPGS, and FA–SA, were developed to improve the chemotherapeutic efficacy of DTX (Figure 1(A)). The average particle size of blank PLN was 141.5 ± 3.1 nm with fairly homogenous dispersity (Table 1). The particle size slightly increased to ∼150 nm after drug loading in the core of the NPs and further increased to ∼165 nm after incorporation of FA–SA on the surface of the NPs. The morphology of NPs was determined by TEM imaging (Figure 1(B)). NPs were perfectly spherical and uniformly distributed and the morphology did not change after drug loading. A grayish shell was observed on the surface of NPs after folate conjugation. It can be attributed to the presence of FA on the surface of NPs. The average diameter of FA-PLN/DTX was slightly higher than that of PLN/DTX as observed in DLS analysis. FA-PLN/DTX showed a high entrapment efficiency (>90%) with an active drug loading of ∼10.3% w/w.

**Table 1.** Dynamic light scattering analysis of nanoformulations.

	Size (nm)	PDI	Zeta potential (mV)	EE (%)	LE (%)
Blank PLN	141.5 ± 3.1	0.125 ± 0.016	–17.9 ± 1.9	–	–
PLN/DTX	148.9 ± 1.7	0.119 ± 0.014	–35.5 ± 1.3	92.5 ± 1.2	11.4 ± 2.1
FA-PLN/DTX	165.1 ± 2.5	0.184 ± 0.019	–29.2 ± 1.6	91.7 ± 1.9	10.3 ± 3.4

### Solid-state characterization

Solid-state characterizations were performed to confirm the successful encapsulation of drug in the NPs. X-ray diffraction (XRD) patterns showed numerous characteristic peaks for free DTX, indicating the crystalline nature of the drug. However, no characteristic peaks were observed in the PLN/DTX or FA-PLN/DTX, indicating the presence of drug in its amorphous form (Supplementary Figure S2). Furthermore, DSC thermogram of DTX showed a sharp endothermic peak at 160 °C corresponding to its melting point. No such endothermic peaks were observed in the formulations, indicating the presence of drug in a molecularly dispersed state (Supplementary Figure S3). Lastly, FTIR spectra also revealed the presence of characteristic peaks of DTX in PLN/DTX and FA-PLN/DTX formulations (Supplementary Figure S4).

### *In vitro* drug release

To simulate the physiological and pathological conditions, a drug release study was performed in phosphate buffered saline (pH 7.4) and acetate buffered saline (pH 5.0) (Figure 1(C)). PLN/DTX and FA-PLN/DTX exhibited controlled drug release at pH 7.4. The presence of FA–SA as the outer layer of PLN slightly influenced the DTX release in both media. For example, ∼65% drug was released from PLN/DTX compared to ∼50% drug release from FA-PLN/DTX after 96 h. In addition, both formulations showed accelerated DTX release in acidic pH conditions.

### Cellular uptake behavior of FA-PLN/DTX

**Figure 2.**
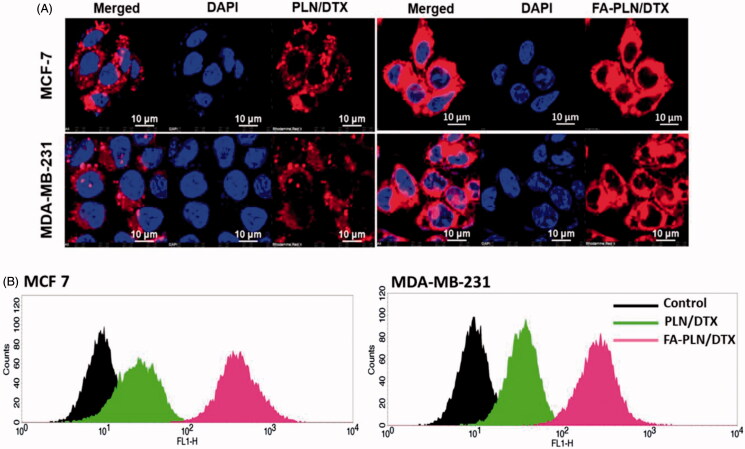
(A) *In vitro* cellular uptake of PLN/DTX and FA-PLN/DTX in MCF-7 and MDA-MB-231 cancer cells. Cellular internalization was observed using a confocal laser scanning microscope (CLSM). Rhodamine-B was used as a fluorescent probe and nuclei were stained with DAPI. (B) Flow cytotmeter based cellular uptake of PLN/DTX and FA-PLN/DTX in MCF-7 and MDA-MB-231 cancer cells.

MCF-7 and MDA-MB-231 cancer cells were incubated with PLN/DTX and FA-PLN/DTX for 2 h. Results clearly showed that fluorescence was mainly observed in the cytoplasmic region, indicating a typical endocytosis-mediated cellular uptake (Figure 2(A)). As expected, FA-PLN/DTX showed significantly higher cellular uptake than that of PLN/DTX in both cancer cells. The targeting efficiency of FA-PLN/DTX was demonstrated by flow cytometer-based cellular uptake (Figure 2(B)). Consistent with the CLSM image, flow cytometer clearly reveals the superior targeting efficiency of FA-PLN/DTX in MCF-7 and MDA-MB-231 cancer cells. The remarkably higher cellular uptake of FA-PLN/DTX was mainly attributed to the FR-based cellular internalization.

### *In vitro* cytotoxicity of FA-PLN/DTX

**Figure 3.**
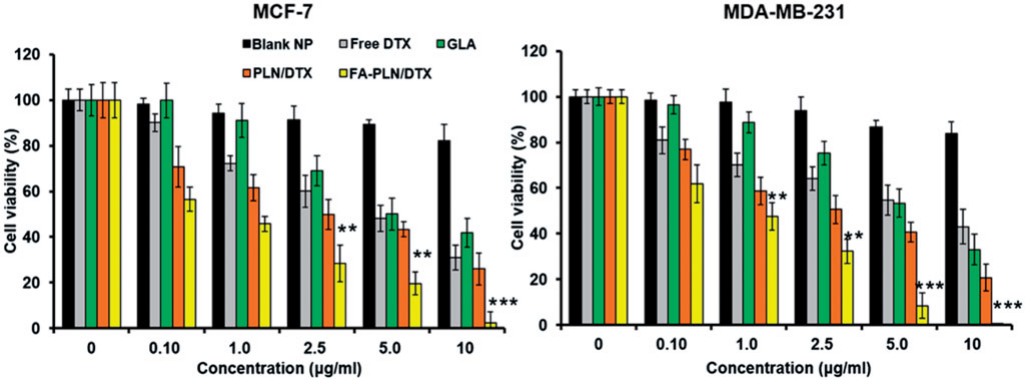
The *in vitro* antitumor activity of nanoformulations in MCF-7 and MDA-MB-231 breast cancer cells. The cells were treated with formulations (0.1–10 µg/mL) for 24 h. The cell proliferation was evaluated by MTT assay. Data are presented as mean ± S.D (*n* = 6).*p < .05, **p < .01, ***p < .001.

In this study, cytotoxic effect of free GLA, DTX, PLN/DTX, and FA-PLN/DTX was studied in MCF-7 and MDA-MB-231 breast cancer cells (Figure 3). All formulations exhibited a dose-dependent cytotoxic effect in both cancer cell lines. When exposed to 10 µg of GLA (loaded in carrier), more than 50% and 40% of MCF-7 and MDA-MB-231 cells were killed, respectively, indicating its antitumor effect. Blank nanocarrier without GLA did not show any effect on the proliferation of cancer cells at all tested concentrations. At equivalent drug concentrations, FA-PLN/DTX exhibited a superior anticancer effect as compared to those of all other formulations. Consistent with these findings, morphological imaging showed significant cell death, cell shrinkage, and membrane blebbing in the FA-PLN/DTX-treated group (Supplementary Figure S5).

### Cell cycle analysis and cell apoptosis

**Figure 4.**
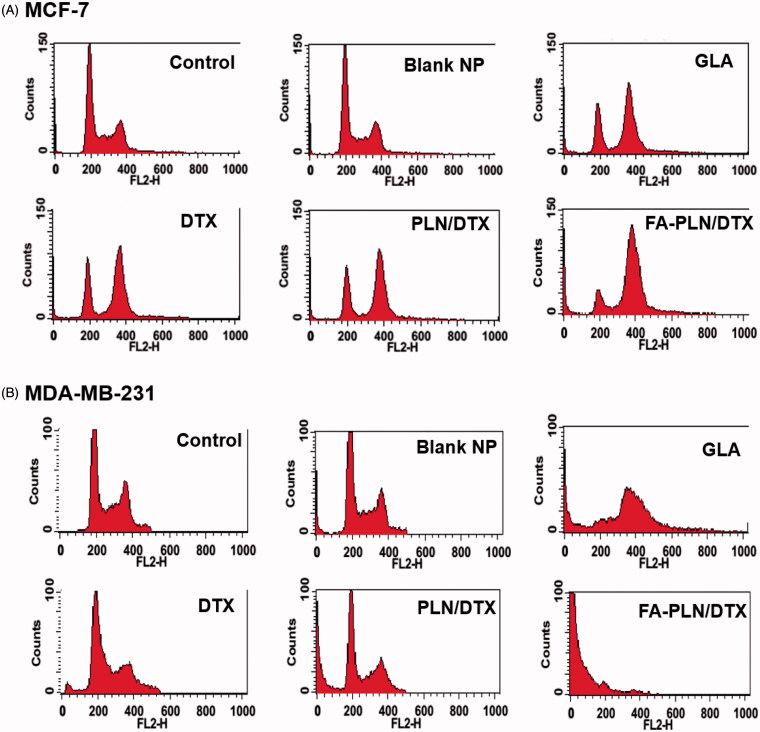
Cell cycle analysis of (A) MCF-7 and (B) MDA-MB-231 cancer cells after exposure to different formulations. The cells were treated with blank NPs without GLA, DTX, GLA, PLN/DTX, and FA-PLN/DTX for 24 h. Cell cycle analysis was performed on a flow cytometer. G0/G1, S, and G2/M show the cell cycle phase, and subG1 refers to the proportion of apoptotic cell.

The cell cycle distribution of MCF-7 and MDA-MB-231 cells after treatment with formulations was also evaluated in this study (Figure 4). As expected, free DTX arrested the cells at G2/M phase of cell cycle. Surprisingly, GLA also induced a typical G2/M phase arrest, indicating its potent anticancer effect. In the FA-PLN/DTX-treated group, ∼70% cells were in the G2/M phase and ∼10% cells were in the sub-G0 phase of the cell cycle. In MDA-MB-231 cells, free DTX induced G2/M phase arrest in ∼20% cell population, whereas GLA treatment induced ∼35% G2/M phase arrest and ∼15% of cells in sub-G0 phase. Importantly, FA-PLN/DTX treated group showed ∼75% cells in sub-G0 phase, indicating its superior therapeutic effect in resistant cancer cells.

**Figure 5.**
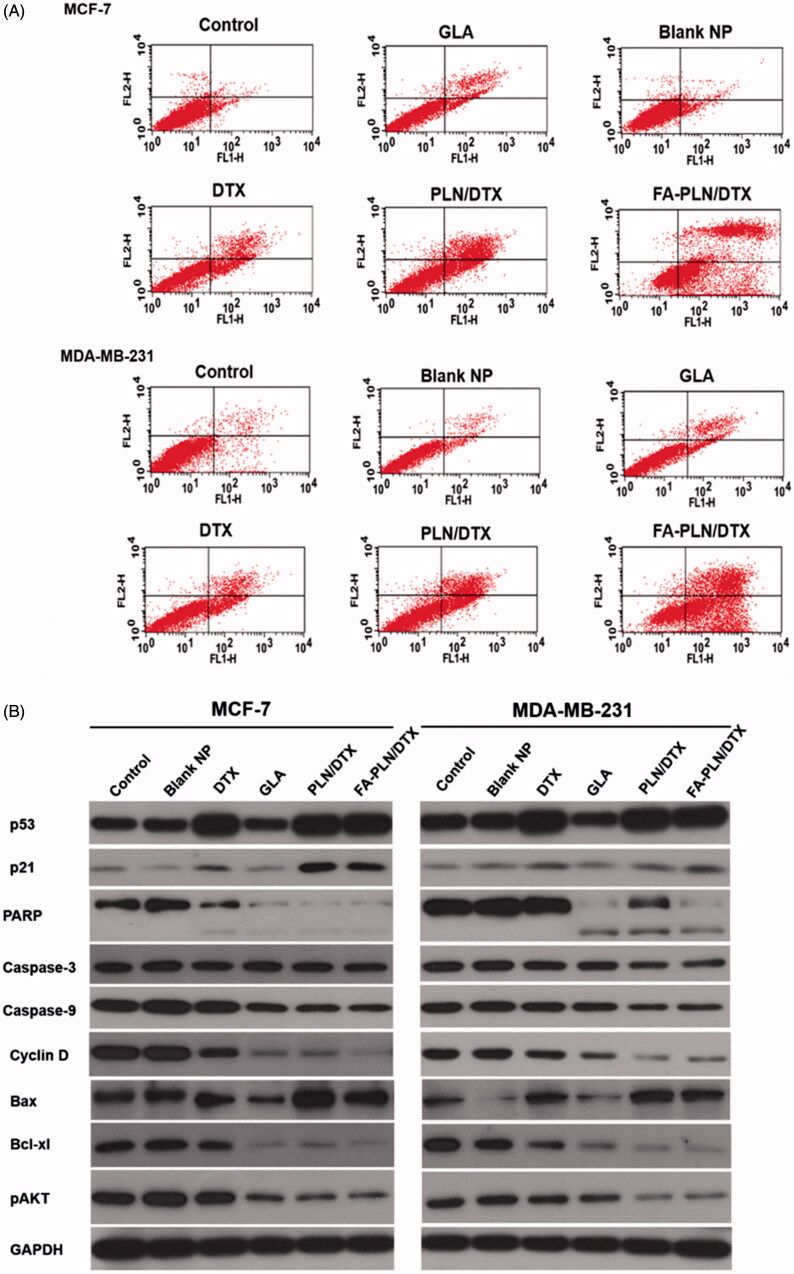
(A) Analysis of apoptotic population of MCF-7 and MDA-MB-231 cells after exposure to formulations. The cells were treated with blank NP without GLA, DTX, GLA, PLN/DTX, and FA-PLN/DTX for 24 h. The apoptotic cell death was assessed by FACS analysis using Annexin V/PI staining. Lower left quadrant indicates viable cells, upper left quadrant indicates the necrotic, and the lower right and upper right quadrants indicate early and late apoptotic cancer cells. (B) Immunoblot analysis of cell cycle, pro- and anti-apoptotic, and AKT-based signaling pathways. The whole-cell lysate was used to analyze the protein expression. Tumor suppressor proteins (p53, p21) were upregulated, cell cycle proteins were downregulated, pro-apoptotic markers (caspase-3/9, PARP) were downregulated, anti-apoptotic protein was downregulated, and AKT signaling pathway was downregulated.

Apoptosis induced in cancer cells after treatment with different formulations was determined by using an Annexin V/FITC and PI staining kit. As shown in Figure 5(A), early apoptosis (Annexin-V^+^ and PI^–^) and late apoptosis (Annexin-V^+^ and PI^+^) were the major mechanisms of cell death in both MCF-7 and MDA-MB-231 cancer cells. The combination of GLA and DTX (PLN/DTX) induced significantly higher apoptosis than any other formulation did in both cancer cells. In MCF-7 cells, PLN/DTX and FA-PLN/DTX induced approximately 65% and 88% apoptosis compared to 42% apoptosis induced by free DTX. Similarly, in MDA-MB-231 cells, approximately 60% and 90% apoptosis were observed for PLN/DTX and FA-PLN/DTX-treated cells, respectively, compared to 30% for free DTX.

### Apoptotic signaling pathways

To understand the molecular mechanisms involved in the anti-proliferative effect of GLA, western blot analysis was performed on MCF-7 and MDA-MB-231 cell lysates (Figure 5(B)). Consistent with the cytotoxicity assay, PLN/DTX and FA-PLN/DTX significantly upregulated the expression of p53 and p21 in both MCF-7 and MDA-MB-231 cancer cells. Furthermore, the expression levels of pro-apoptotic markers (PARP, caspase-3 and caspase-9) and an anti-apoptotic marker (Bcl-xl) were analyzed. Combination of GLA and DTX significantly downregulated the expression levels of PARP, caspase-3, and caspase-9. Moreover, results indicated that PARP polypeptide was cleaved from 116-kDa fragments into smaller 89-kDa fragments. Bcl-xl (member of Bcl-2 family), which is an important regulator of apoptosis, was markedly downregulated by PLN/DTX and FA-PLN/DTX treatment. In the present study, DTX showed negligible effect on the phosphorylation of AKT in both cancer cells; however, GLA significantly inhibited the phosphorylation of AKT. Consistently, PLN/DTX and FA-PLN/DTX significantly downregulated the expression levels of pAKT.

### *In vivo* antitumor effect of FA-PLN/DTX

**Figure 6.**
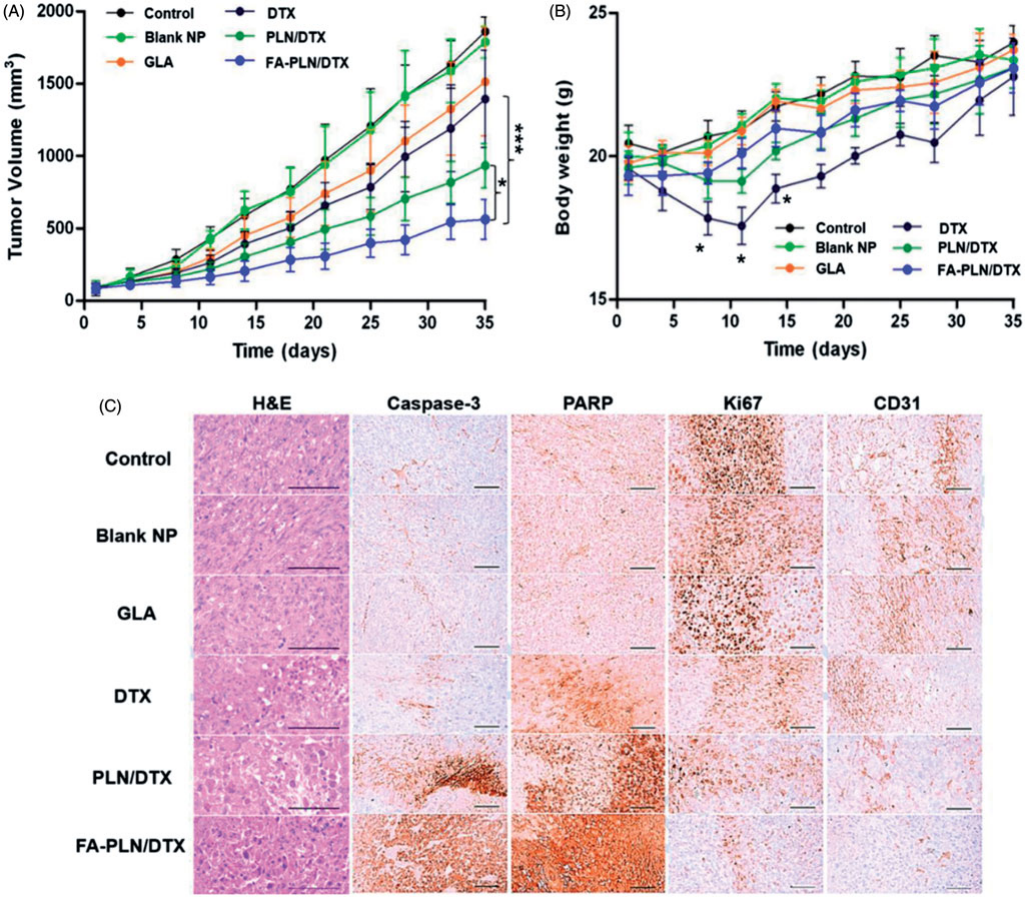
*In vivo* antitumor efficacy of FA-PLN/DTX in MDA-MB-231 xenograft nude mice. (A) Tumor volume changes in mice treated with developed formulations. (B) Body weight changes. (C) Histological and immunohistochemical analysis after different treatments. The formulations were administered via the tail vein at a fixed dose of 5 mg/kg on days 1, 4, and 7. Data are presented as mean ± S.D (*n* = 8). *p < .05, **p < .01, ***p < .001.

The *in vivo* therapeutic efficacy of nanoformulations was evaluated in MDA-MB-231 cell-bearing tumor xenograft mice model. The mice were intravenously injected free DTX, GLA, PLN/DTX, and FA-PLN/DTX at a fixed dose of 5 mg/kg. As shown in Figure 6(A), FA-PLN/DTX treatment resulted in remarkable tumor regression. In contrast, tumors continued to grow in free DTX and GLA-treated groups. As shown in Figure 6(B), mice in the free DTX-treated group lost more than 20% of body weight, indicating the severe toxic effect of DTX. In contrast, mice in PLN/DTX and FA-PLN/DTX treatment groups did not experience any body weight loss.

Histological analysis (H&E staining) of extracted tumors further showed the superior apoptosis-inducing potential of FA-PLN/DTX (Figure 5(C)). H&E staining showed that FA-PLN/DTX induced severe necrosis of tumor cells across a large area of tumor section and dead cells with condensed nuclei were seen. In contrast, free DTX or GLA alone did not cause significant damage to the tumor tissues. The untreated control group showed that tumor cells had large nuclei with intact morphology. The levels of caspase-3 and PARP are markers of apoptosis in tumor models. The mice treated with blank NP without GLA, free DTX, GLA, PLN/DTX, and FA-PLN/DTX showed 9.36, 31.82, 10.89, 53.72, and 77.47% of caspase-3 immunolabeled cells, respectively, whereas the control group mice showed 9.85% (Supplementary Table S1). Similarly, FA-PLN/DTX showed the highest PARP immunolabeled cells (∼80%). The number of CD31 + and Ki-67 + cancer cells was significantly lower in the FA-PLN/DTX-treated group than in other groups. Overall, results of the current study provide substantial evidence of the superior antitumor efficacy of FA-PLN/DTX *in vivo*.

## Discussion

Nanomedicine offers an attractive approach for antitumor drug delivery and holds a great potential in cancer treatment. In this study, novel PUFA-based lipid NPs comprising TPGS and FA-SA were developed to improve the chemotherapeutic efficacy of DTX. The NPs were prepared by hot homogenization followed by sonication method. The average particle size of FA-PLN/DTX was ∼165 nm. We suggest that this particle size <200 nm and narrow size distribution could benefit in cancer targeting applications. It has been reported that the nanosized particles could accumulate in the tumor tissues by virtue of enhanced permeation and retention (EPR) effect (Choi et al., 2015). Therefore, it can be suggested that narrow size distribution and sub 200 nm size of NPs developed in this study will benefit in the cancer targeting applications. Zeta potential is important for the colloidal stability of particles and influences their circulation time, metabolism, clearance and immune response. A strong zeta potential of –25 or +25 mV would impart a high colloidal stability ( Ramasamy et al., 2017). Moreover, negative surface charge can help to avoid the unnecessary plasma protein adhesion and RES-based clearances.

An *in vitro* drug release study was performed in PBS and ABS to simulate the *in vivo* conditions. PLN/DTX and FA-PLN/DTX exhibited a controlled release of DTX in both pH environments. The presence of FA on the outer layer of NPs significantly helped to control the release of drug in the respective media. Absence of initial burst release indicates complete incorporation of drug in the core of NPs and shows that drug was not present on the surface. The ability of nanocarriers to release drugs in a controlled manner in physiological conditions and accelerate drug release in acidic conditions suggests their application in tumor targeting after internalization into the cells (Kim et al., 2014; Ramasamy et al., 2014). Furthermore, this can effectively prevent against the release of drugs in the systemic circulation and the associated side effects. Both type of NPs exhibited excellent storage stability until 3 months. The excellent colloidal stability can be mainly attributed to the stearic stability provided by outer PEG shell on the NP surface.

Multidrug resistant (MDR) cancer cells efflux out anticancer drugs through P-glycoprotein (P-gp). We hypothesized that the presence of GLA and TPGS in NPs will effectively inhibit the P-gp, thereby enhancing the chemotherapeutic efficacy of DTX. As expected, FA-PLN showed significantly higher cellular uptake than that of PLN in both cancer cells. This higher cellular uptake of FA-PLN was attributed to its specific binding to the FRs on the cell membrane (Sriraman et al., 2016). A specific folate-receptor mediated endocytosis mechanism could have resulted in the higher accumulation of the NPs in the cytoplasmic region, as shown by the bright red fluorescence (Esfandyari-Manesh et al., 2016). The higher intracellular accumulation of NP and presence of GLA and TPGS could have helped the anticancer drugs to escape from the efflux action of P-gp.

Several studies have reported that PUFAs such as GLA possess potent antitumor effect, both *in vitro* and *in vivo*. PUFAs induce the cytotoxic effects by increasing free radical generation, lipid peroxidation, changing the membrane composition, and mitochondrial dysfunction. FA-PLN/DTX exhibited a superior anticancer effect as compared to those of all other formulations. This could be due to the fact that PLN and DTX pass through the cell membrane via passive diffusion, while FA-PLN are internalized in the cancer cells by the high affinity of targeting ligand (FA) to the FRs in the cancer cells. These results suggest that GLA has synergistic potential that enhanced the activity of encapsulated drug. Cancer cells have deficient Δ6 and Δ5 desaturase activity and, therefore, accumulate low amount of GLA or EPA compared to that in healthy cells. PUFA can generate free radicals that can decrease the activity of desaturases, which is a defense mechanism adopted by tumor cells (Youn & Lee, 2014). Therefore, presence of PUFA could compromise the defense mechanism of tumor cells, and synergistically enhance the therapeutic efficacy of DTX in cancer treatment.

The effect of GLA on enhancing the chemotherapeutic efficacy of DTX was further confirmed by cell cycle analysis. There are checkpoints, at the entrance of cells into the next phase, in the cell cycle that control the process of cell division. DTX binds to tubulin dimer present in microtubules and induces G2/M phase arrest in rapidly dividing cells, eventually resulting in apoptosis. Results clearly showed that GLA effectively arrested the cells at G2/M phase with substantial presence in sub-G0 phase, indicating high apoptosis. Therefore, combination of GLA and DTX in a single carrier and a targeting ligand on the surface of the carrier will result in significant inhibition of the proliferation of cancer cells. Moreover, targeting ligand could have helped in actively transporting the therapeutics across the cell membrane, resulting in increased intracellular concentrations. Taken together, these results indicated that FA-PLN/DTX inhibited the proliferation and growth of tumor cells by inducing a G2/M phase arrest and cell apoptosis.

As mentioned above, presence of PUFA enhanced the anticancer efficacy of DTX in both cancer cells, and targeting ligand actively transported the NPs across the cell membrane and increased the intracellular concentrations, resulting in a synergistic effect. Western blot analysis was performed to understand the molecular mechanisms involved in the anti-proliferative effect of PUFA. It has been reported that p53-mediated transcription is necessary to maintain the arrest and the p21 protein inhibits cyclin E-Cdk2 or cyclin D-Cdk4 complexes, thereby, suppressing the Rb/E2F pathway and cell death (Sundaramoorthy et al., 2016). Chemotherapeutic agents inhibit cancer cell growth by upregulating the tumor suppressor genes such as p53 and p21. FA-PLN/DTX remarkably up-regulated p53 and p21 expression in MCF-7 and MDA-MB-231 cancer cells. Results of the present study suggested that DTX and PUFA exert anticancer effect through cell cycle arrest at G2/M phase. Internally, cyclins play an important role in controlling the apoptosis. G2/M checkpoint ensures that cells do not enter mitosis phase, resulting in death. Our results indicated that FA-PLN/DTX markedly downregulated the expression of cyclin D, which is an important regulatory protein. Furthermore, the synergistic combination of PUFA and DTX significantly downregulated the expression levels of PARP, caspase-3, and caspase-9. Data revealed a cleavage of the PARP polypeptide from 116-kDa fragments into smaller 89-kDa fragments. The cleavage of PARP enzyme is a reliable marker for determining the onset of cellular apoptosis. Cleavage of PARP after treatment with NP formulations indicated apoptosis in treated cells.

Bcl-2 promotes cell survival by interfering with activation of the cytochrome c/Apaf-1 pathway through stabilization of the mitochondrial membrane. The downregulation of Bcl-xl (a member of Bcl-2 family) may cause mitochondrial dysfunction, initiate the proteolytic processing, and activate several caspases, resulting in apoptosis and cell death (Vander Heiden & Thompson, 1999). Breast cancer cells are generally associated with AKT signaling pathways that upregulate cell proliferation and suppress cell death mechanism (Manning & Cantley, 2007). FA-PLN/DTX significantly downregulated the expression levels of pAKT. Results of the present study corroborate with the previous findings that persistent blockage of AKT signaling pathway by PUFA is directly related with their tumor inhibitory effects. The effect of PUFA on AKT phosphorylation could be attributed to the recruitment of cytosolic AKT to the cell membrane surface where it interacts with PDK1, altering the accessibility to PIP3 and PTEN.

The therapeutic performance of nanoformulations was evaluated using MDA-MB-231 cancer cells-bearing tumor xenograft mouse model. The results clearly showed the remarkable tumor regression effect of FA-PLN/DTX. The remarkable tumor inhibitory effect of FA-PLN/DTX (∼75%) could be attributed to two important factors; first, tumor targeting efficiency of FA-PLN/DTX towards FR overexpressing MDA-MB-231 tumors, which could have led to efficient internalization of carriers and thereby enhanced accumulation of anticancer agents. Second, synergistic potential of GLA with DTX, which enhanced the pharmacological activity of encapsulated drug (DTX). As mentioned above, GLA can generate free radicals that decrease the activity of desaturases, which are a defense mechanism used by tumor cells, leading to synergistic enhancement of the therapeutic efficacy of DTX in cancer treatment. Antitumor drugs are often reported to induce severe side effects in healthy cells. Body weight of the tumor-bearing mice was noted at specified time points as a safety index. Treatment with formulations did not result in significant body weight change, indicating that drug encapsulation in a nanocarrier could improve its safety profile and effectively reduce its side effects. H&E staining showed that FA-PLN/DTX induced severe necrosis of tumor cells across the tumor sections. Moreover, it showed dead cells with condensed nuclei. The levels of caspase-3 and PARP provide an indication of apoptosis in tumor models. The FA-PLN/DTX group showed the highest caspase-3 and PARP-immunolabeled cells (∼80%). CD-31 and Ki67 levels were analyzed as markers for angiogenesis and tumor proliferation. The number of CD31 + and Ki-67 + cancer cells was significantly lower in the FA-PLN/DTX-treated group than in the other groups. Overall, results of the current study provide substantial evidence of the superior antitumor efficacy of FA-PLN/DTX *in vivo*.

## Conclusion

In summary, we designed and developed novel PUFA-based FA-conjugated lipid NPs that could efficiently target and treat human breast tumor xenograft *in vivo*. The developed FA-PLN/DTX showed high payload capacity with controlled drug release characteristics. The presence of PUFA synergistically enhanced the anticancer efficacy of DTX in both MCF-7 and MDA-MB-231 cancer cells by inducing a G2/M phase arrest and cell apoptosis. FA-PLN/DTX remarkably downregulated the expression levels of pro-apoptotic and anti-apoptotic markers, and blocked the phosphorylation of AKT signaling pathways. In addition, *in vivo* study showed that NPs had excellent antitumor efficacy with minimal side effects. Based on our findings, FA-PLN/DTX could effectively improve the therapeutic efficacy of anticancer agents in cancer therapy and could be a promising therapeutic strategy for cancer treatment. Further studies are necessary to fully exploit these findings to achieve clinical translation.

## Supplementary Material

IDRD_Kim_et_al_Supplemental_Content.docx
