# Supplementary material for: Polyunsaturated fatty acid-based targeted nanotherapeutics to enhance the therapeutic efficacy of docetaxel
Source: Drug Deliv. 2017 Sep 9;24(1):1262–72. doi: 10.1080/10717544.2017.1373163 (PMC8241009; doi:10.1080/10717544.2017.1373163)
Supplement: IDRD_Kim_et_al_Supplemental_Content.docx [file IDRD_A_1373163_SM8041.docx]

**Supplementary Information**

**Polyunsaturated fatty acids-based targeted nanotherapeutics to enhance the therapeutic efficacy of docetaxel**

Thiruganesh Ramasamy,^a,b^ Pasupathi Sundaramoorthy,^c,d^ Hima Bindu Ruttala,^a^ Yongjoo Choi,^a^ Woo Hyun Shin,^a^ Jee-Heon Jeong,^a^ Sae Kwang Ku,^e^ Han-Gon Choi,^f^ Hwan Mook Kim,^c^ Chul Soon Yong,^a,*^ and Jong Oh Kim^a,*^

^a^College of Pharmacy, Yeungnam University, 214-1, Dae-dong, Gyeongsan, 712-749, Republic of Korea

^b^Center for Ultrasound Molecular Imaging and Therapeutics, Department of Medicine, University of Pittsburgh, Pittsburgh, PA 15213, USA

^c^Gachon Institute of Pharmaceutical Sciences, Gachon University, Incheon 406-840, Republic of Korea

^d^Division of Hematologic Malignancies & Cellular Therapy, Duke University Medical Center, Durham, North Carolina 27705, USA

^e^College of Korean Medicine, Daegu Haany University, Gyeongsan, 712-715, Republic of Korea

^f^College of Pharmacy**, Institute of Pharmaceutical Science and Technology,** Hanyang University, 55, Hanyangdaehak-ro, Sangnok-gu, Ansan 426-791, Republic of Korea

**^*^**Corresponding authors:

**Jong Oh Kim**; College of Pharmacy, Yeungnam University, 214-1, Dae-dong, Gyeongsan, 712-749, Republic of Korea

E-mail: jongohkim@yu.ac.kr; Tel: +82-53-810-2813; Fax: +82-53-810-4654

**Chul Soon Yong**; College of Pharmacy, Yeungnam University, 214-1, Dae-dong, Gyeongsan, 712-749, Republic of Korea

E-mail: csyong@ynu.ac.kr; Tel: +82-53-810-2812; Fax: +82-53-810-4654

(A)


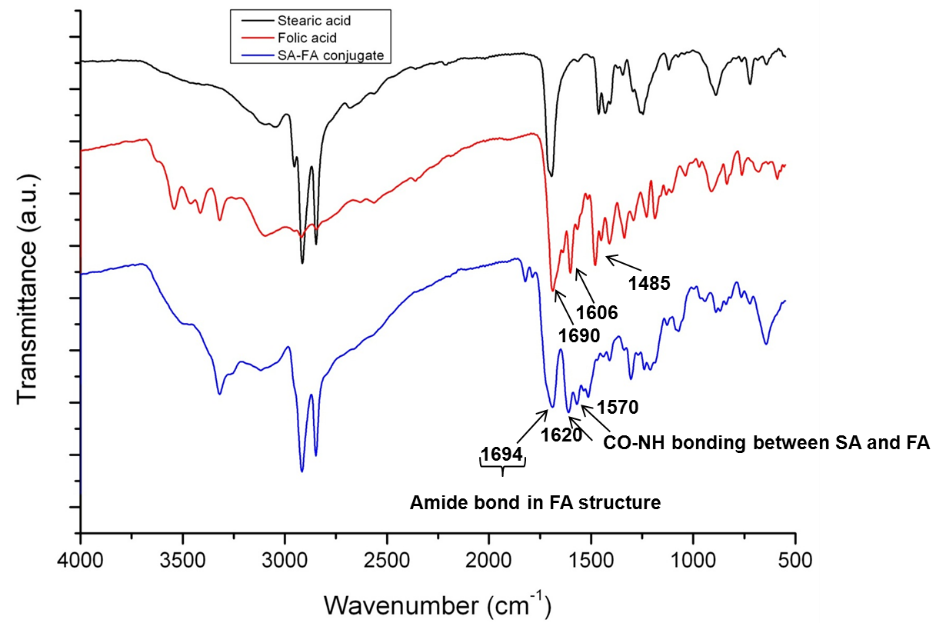


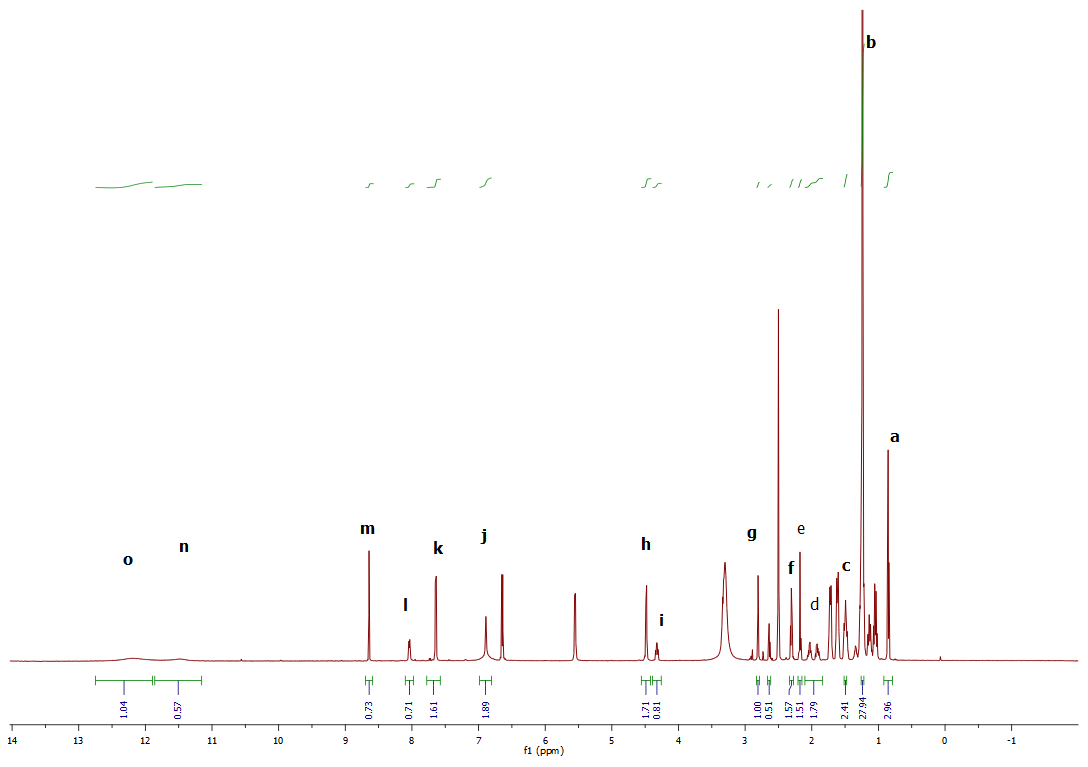
(B)

**Figure S1**. (A) ^1^H NMR analysis of stearic acid-folic acid conjugate (FA-SA). (B) FTIR analysis of FA-SA.

For this purpose, FA-SA conjugate was prepared. The successful formation of FA-SA conjugate was first confirmed by FTIR analysis (**Fig. S1A**). The carbonyl (C=O) and amine (N−H) groups present in the amide linkage exhibited bands at 1620 and 1570 cm^−1^. Bands at 1485 and 1606 cm^−1^ may be due to the stretching vibrations of C=C in the backbone of the aromatic ring present in folic acid. The chemical conjugate was further analyzed by ^1^H NMR spectra. In the ^1^H NMR spectrum of FA-SA, peak at 2.82 ppm corresponds to the amide bond linkage between FA and SA. The peaks at 6.95 and 7.68 ppm were attributed to the aromatic protons of folate moiety and peaks at 1.25 ppm were assigned to methylene protons of SA (**Fig. S1B**). Methylene group of folate residue was confirmed at 4.5 ppm and hydroxyl group was observed at 11.5 and 12.28 ppm, respectively.


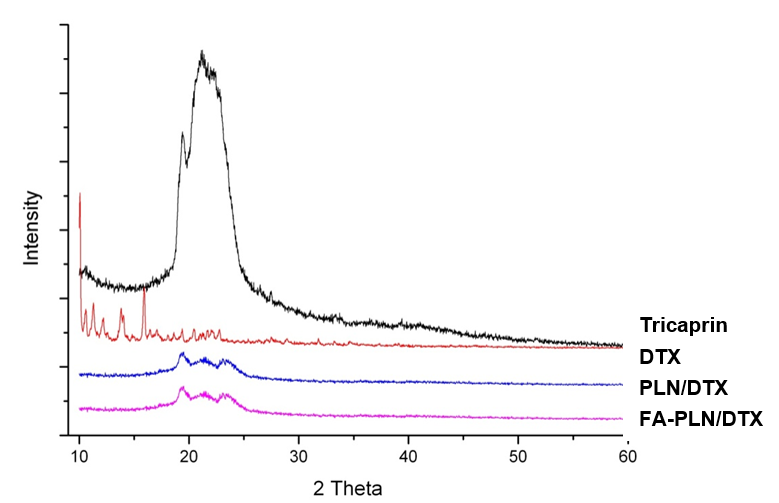


**Figure S2**. X-ray diffraction (XRD) analysis of tricaprin, DTX, PLN/DTX, and FA-PLN/DTX.


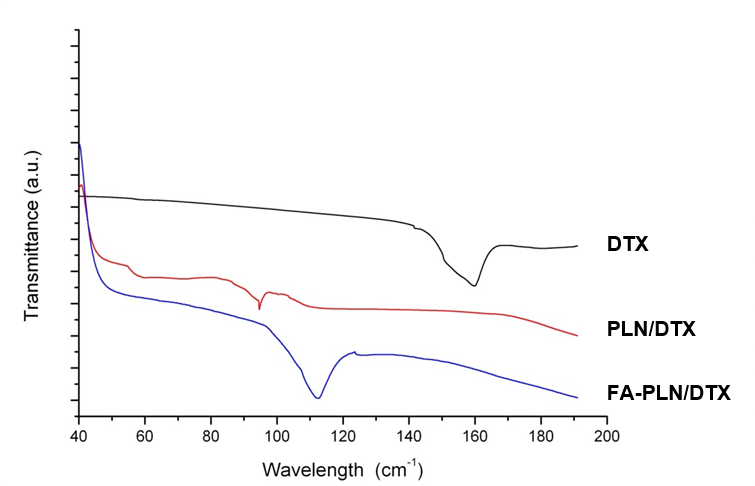


**Figure S3**. Dynamic scanning colorimetry (DSC) analysis of DTX, PLN/DTX, and FA-PLN/DTX.


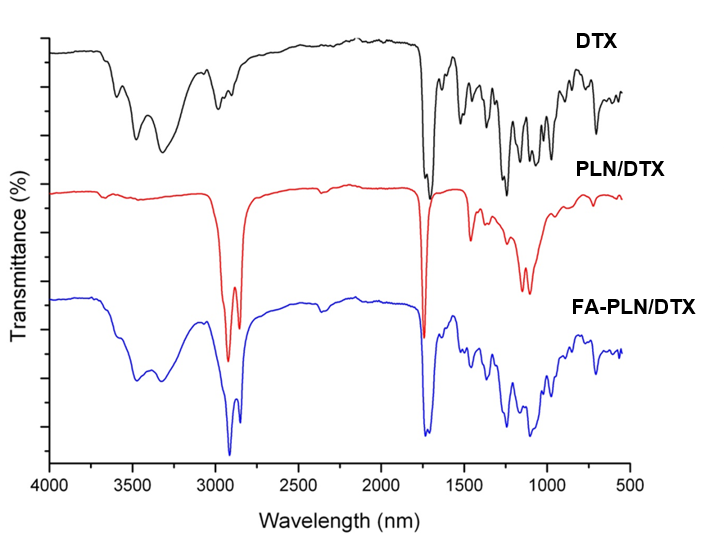


**Figure S4**. Fourier transform-infra red (FTIR) analysis of DTX, PLN/DTX, and FA-PLN/DTX.


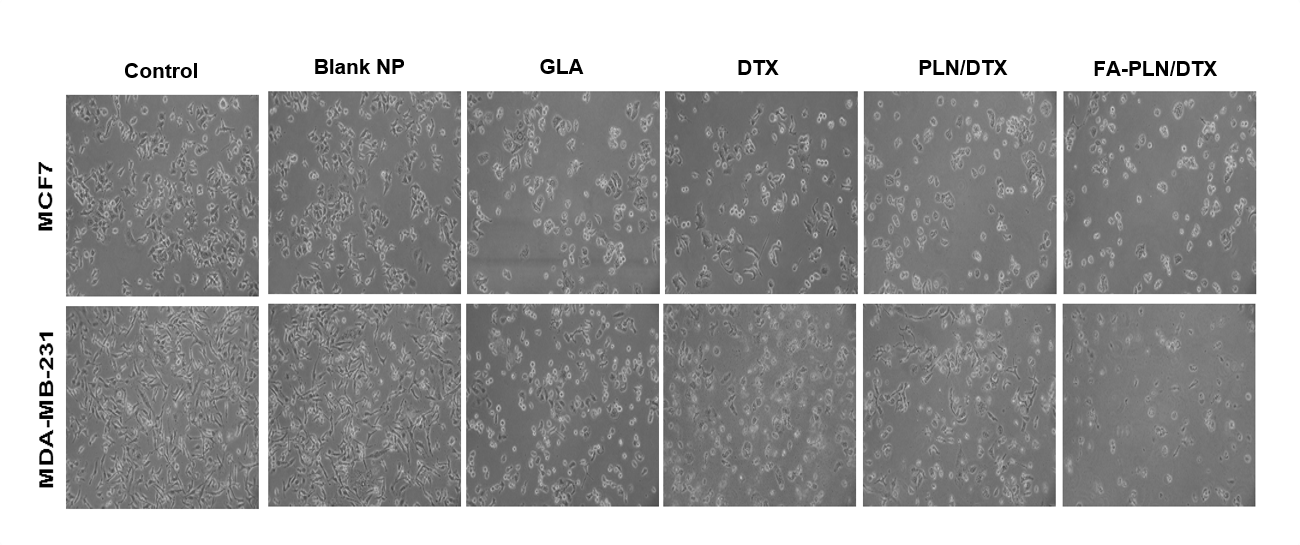


**Figure S5**. Phase contrast morphology analysis MCF-7 and MDA-MB-231 cancer after exposure with respective formulations for 24h.

#### Table S1. Histomorphometrical analysis of tumor masses, taken form xenograft athymic nude mice

| Groups | | Tumor cell volumes  (%/mm^2^ of tumor mass) | Immunolabeled cell percentages (%/mm^2^ of tumor mass) | | | |
| --- | --- | --- | --- | --- | --- | --- |
|  |  |  | Caspase-3 | PARP | Ki-67 | CD31 (PECAM-1) |
|  | |  |  |  |  |  |
| G1 | Control | 80.74 ± 11.55 | 9.85 ± 3.67 | 11.39 ± 4.35 | 76.20 ± 12.18 | 47.49 ± 13.13 |
| G2 | Blank NP | 79.64 ± 10.34 | 9.36 ± 5.33 | 11.97 ± 5.09 | 75.88 ± 10.98 | 46.35 ± 13.42 |
| G3 | GLA | 78.45 ± 11.06 | 10.89 ± 2.89 | 12.76 ± 2.89 | 73.86 ± 10.82 | 44.30 ± 11.62 |
| G4 | DTX | 57.30 ± 11.83^abc^ | 31.82 ± 5.17^ghi^ | 36.34 ± 11.14^abc^ | 47.09 ± 10.65^abc^ | 24.24 ± 5.43^ghi^ |
| G5 | PLN/DTX | 36.81 ± 10.16^abcd^ | 53.72 ± 8.61^ghij^ | 57.58 ± 12.24^abcd^ | 28.62 ± 5.55^abcd^ | 15.61 ± 3.71^ghik^ |
| G6 | FA-PLN/DTX | 17.52 ± 4.89^abcde^ | 77.47 ± 11.68^ghijl^ | 78.41 ± 10.22^abcde^ | 15.97 ± 5.05^abcdf^ | 7.87 ± 1.82^ghijl^ |

Values are expressed as mean ± SD of six tumor mass histological fields

PARP = Cleaved poly(ADP-ribose) polymerase; PECAM-1 = Platelet endothelial cell adhesion molecule 1 (CD31)

^a^ p<0.01 as compared with G1 by LSD test ^g^ p<0.01 as compared with G1 by MW test

^b^ p<0.01 as compared with G2 by LSD test ^h^ p<0.01 as compared with G2 by MW test

^c^ p<0.01 as compared with G3 by LSD test ^i^ p<0.01 as compared with G3 by MW test

^d^ p<0.01 as compared with G4 by LSD test ^j^ p<0.01 and ^k^ p<0.05 as compared with G4 by MW test

^e^ p<0.01 and ^f^ p<0.05 as compared with G5 by LSD test ^l^ p<0.01 as compared with G5 by MW test
